# Proximate, mineral and anti-nutrient compositions of oat grains (*Avena sativa*) cultivated in Ethiopia: implications for nutrition and mineral bioavailability

**DOI:** 10.1016/j.heliyon.2021.e07722

**Published:** 2021-08-05

**Authors:** Getaneh Firew Alemayehu, Sirawdink Fikreyesus Forsido, Yetenayet B. Tola, Minbale Adimas Teshager, Addisu Alemayehu Assegie, Endale Amare

**Affiliations:** a Department of Post-Harvest Management, Jimma University, Jimma, Ethiopia; b Department of Chemistry, Debre Markos University, Debre Markos, Ethiopia; c Food Science and Nutrition Research Directorate, Ethiopian Public Health Institute, Addis Ababa, Ethiopia; d Department of Material Science and Engineering, College of Science, Bahir Dar University, Bahir Dar, Ethiopia

**Keywords:** Anti-nutrient, Mineral, Mineral bioavailability, Oats, Proximate composition

## Abstract

Oat (*Avena sativa*) is an underutilized cereal grain in Ethiopia from the *Poaceae* grass family. This study aimed to investigate the proximate, mineral, and anti-nutrient composition of three landrace varieties commonly used in certain districts of the country and compare them with two improved varieties of oats in Ethiopia. The proximate and mineral composition was determined using the Association of Official Analytical Chemists (AOAC) standard methods. Phytate and tannin contents were determined using the spectroscopic method, and oxalate was analyzed using HPLC. The bioavailability of minerals was also estimated. Results showed significant (p < 0.05) differences in proximate, mineral, and anti-nutrient compositions among studied varieties. The moisture, crude protein, crude fat, crude fiber, ash, and total carbohydrate contents were in the range between 8.5-9.8, 11.9–15.8, 6.7–10.3, 2.1–3.5, 1.2–1.3, and 72.6–74.3 g/100 g DM, respectively. Iron, copper, zinc, magnesium, calcium, and potassium contents were 2.5–3.0, 0.2–0.4, 1.6–2.0, 62.4–89.1, 44.0–102.7, and 241.7–258.3 mg/100 g DM, respectively. The oxalate, tannin, and phytate contents ranged from 28.2-71.4, 38.8–51.5, and 269.6–293.0 mg/100 g DM, respectively. Except for a few varieties of oats, the molar ratios were below the critical values. Results showed that both the landraces and improved varieties studied are an excellent source of valuable nutrients. Thus, the production and utilization of this crop in a few geographical locations and communities should be further encouraged in the rest areas of the country to benefit from this underutilized but nutritious crop.

## 1 Introduction

Oats (*Avena sativa*) have been grown for thousands of years, mainly as an animal feed crop, but during the 19^th^ century, oats got acceptance as a part of the human diet (Hareland and Manthey, 2003). Substantial quantities of oats are produced annually in highland and midland areas of Ethiopia. Except in a few areas, oats are mostly used for animal feed. However, its significance as human food is unknown in the rest of the country (Mosissa et al., 2018).

Commercially available oats in different parts of the world are well known for their nutritional benefits due to their high composition of lipids, soluble fiber, unsaturated fatty acids, essential amino acids, minerals, vitamins, and avenathramide, an antioxidant found only in oats (Youssef et al., 2016; Sangwan et al., 2014). According to various literature sources, oat is high in oil content and has a well-balanced amino acid composition when compared to other cereal grains (Decker et al., 2014; Peltonen-Sainio et al., 2003; Mäkinen et al., 2017).

A more diverse food supply would result from utilizing the vast reservoir of minor and underutilized plants (Frison et al., 2005). There are numerous crop varieties, but only a limited fraction of them are scientifically studied and documented (Fanzo et al., 2013). Oat varieties studied in this work are an excellent example of this. They are consumed for years as a staple food only in the Northwest parts of Ethiopia. According to prior research, only five districts in the country have been producing three local varieties of oats for nearly a century (Getaneh et al., 2021). The same crop but different landraces are used as livestock feed in other parts of the country. The crop known locally as “Engido”, grows in less fertile soil and is more resistant to drought and pests than many other crops grown in the same area.

Oat-based meals are culturally processed into varieties of foods in the study districts. The most popular oat-based foods include *Injera* (an Ethiopian national dish made from leavened cereal flour that gives a thin flatbread with a bubbly top and a flat bottom), *Kitta* (unleavened flat thin bread), *Anebabero* (a type of bread made by layering two loaves of leavened bread on top of each other), *Enket* (oat-based unique food in the region, made from toasted-crushed oat and *Utrica simensis*), porridge, gruel, and *Tella,* a traditional alcoholic beverage (Getaneh et al., 2021). Even though oat is a staple food crop in the study area, no scientific studies have been conducted to determine the nutritional values and other compositions of commonly used oat varieties. Therefore, the goal of this study was to determine the proximate composition, mineral content, and anti-nutrient content of landrace and improved varieties. Besides, estimation of mineral bioavailability using molar ratios was assessed. Ultimately, the information generated from this work will help to commercialize and broaden the crop's use in different regions of the country and elsewhere in the world.

## 2 Materials and methods

### 2.1 Sample collection

Two recently (2019) released oats cultivars, Goslin and Soresi, were collected from Adet Agricultural Research Center and three local varieties of oats (black-colored, white-colored, and yellow-colored) were collected from the Gozamin district of East Gojjam, Ethiopia. The three local varieties are the only landraces widely grown and used by the specific community. East Gojjam is the only zone that is broadly producing and utilizing the crop for food purposes. According to the survey study we conducted in the area, the three landraces differed in their color due to 100 years of continuous selection and crop improvement practices by the local community.

### 2.2 Sample preparation

The grains were sorted and cleaned manually to remove foreign matters, cracked and broken seeds, and then dehulled using local wood-made pestle and mortar to remove the husk before use. The grains were then pulverized using an electric grinder to pass through a 0.05 mm sieve, packed in polyethylene bags, and stored at 4 °C until further analysis.

### 2.3 Analysis of proximate compositions

#### 2.3.1 Moisture content

The moisture content of the flour sample was determined using the AOAC (2000) official method 925.10. Firstly, the weight of the cleaned and dried aluminum dish was measured (W_1_) and 5 g of sample flour was transferred and weighed with its content (W_2_). The dish and its contents were then heated in a 105 °C oven for 3 h (LABQUIP, LEICESTER LE67 5FT, England). After drying, the sample was cooled in desiccators (CSN-SIMAX) for 30 min and reweighed until a constant weight is achieved (W_3_). The moisture content was then calculated using the weight loss by difference, as shown in Eq. (1).

(1) $$\text{Moisture}\;\left(\text{\%}\right)=\frac{{\text{W}}_{2}-{\text{W}}_{3}}{{\text{W}}_{2}-{\text{W}}_{1}}\times 100$$

where; W_1_ - the weight of the dish, W_2_ - the weight of the dish and sample before drying, and W_3_ - the weight of the dish and sample after drying.

#### 2.3.2 Crude protein content

The crude protein content of the sample flour was determined using an automatic Velp Scientifica Kjeldahl analyzer instrument (UDK 159) following AOAC (2000) official method 979.06. Before digestion, 1.0 g of sample flour was treated with catalysts (CuSO_4_.5H2O and K_2_SO_4_). Ammonia was distilled off during 60 min of digestion of ammonium sulfate at 420 °C in 12 ml concentrated sulfuric acid and reagents 50 ml NaOH, 30 ml H_3_BO_3_, and 50 ml distilled water, which was then automatically titrated with standard hydrochloric acid (0.2 N). Eq. (2) was used to determine the total nitrogen content.

(2) $$\text{Nitrogen}\;\left(\text{\%}\right)=\frac{{\left({\text{V}}_{\text{s}}-{\text{V}}_{\text{b}}\right)}_{\text{HCl}}\times {\text{N}}_{\text{HCl}}\times 14.01}{\text{gram of sample}}\times 100$$

where; V_s_ - the volume of the acid consumed by the sample, V_b_ - the volume of the acid consumed by the blank, N_HCl_ - the normality of the HCl, 14.01- molecular weight of nitrogen.

Crude protein percentage was determined by multiplying the percentage of nitrogen using a conversion factor of 5.36 (Nielsen, 2010). The crude protein percentage was calculated as shown in Eq. (3).

(3) Crude protein(%)=%N×5.36

#### 2.3.3 Crude fat content

The amount of crude fat was determined using the Soxhlet extraction method, as described in AOAC (2000) official method 920.39. Briefly, 2 g of sample (W_1_) was transferred into a thimble and covered with fat-free cotton, and then fitted into the Soxhlet extraction apparatus. A weight of pre-cleaned and dried extraction cylinder was measured (W_2_) and 50 ml of diethyl ether was added to extract the crude fat. The extraction continued for 4 h and then dried in an oven (Blast Air Oven, DHG-9240A, China), adjusted to 70 °C for 30 min. It was then cooled in desiccators for 30 min. The combined weight of the extraction cylinder and extract was measured (W_3_) and crude fat content was determined according to Eq. (4).

(4) $$\text{Crude fat}\;\left(\text{\%}\right)=\frac{{\text{W}}_{3}-{\text{W}}_{2}}{{\text{W}}_{1}}\times 100$$

where; W_3_ - the weight of the extraction cylinder and crude fat, W_2_ - the weight of the extraction cylinder, and W_1_ - the weight of the sample.

#### 2.3.4 Crude fiber content

Crude fiber content was determined based on AOAC (2000) official method 962.09. One gram of sample (W_1_) was boiled for 40 min in 50 ml dilute sulfuric acid (2.5 %) and then, the acid was drained using a vacuum pump. After washing with distilled water, the residue was boiled for 40 min in 50 ml of 2.5 % sodium hydroxide. After that, it was washed with once 20 ml of 99.8 % ethanol, twice with 20 ml of diethyl ether, and three times with 20 ml of acetone. The insoluble residue (crude fiber and ash) was dried in an oven (Blast Air Oven, DHG-9240A, China) and weighed (W_2_). This residue was burned at 550 °C for 3 h (W_3_) in a furnace (Nabertherm, D-6072 Dreieich, Germany), and the crude fiber percentage was calculated using Eq. (5).

(5) $$\text{Crude fiber}\;\left(\text{\%}\right)=\frac{{\text{W}}_{2}-{\text{W}}_{3}}{{\text{W}}_{1}}\times 100$$

where; W_1_ - the weight of the sample, W_2_ - the weight of dried insoluble residue (crude fiber + ash), and W_3_ - the weight of burned residue (ash).

#### 2.3.5 Ash content

The ash content was determined after the removal of organic matter by dry ashing according to AOAC (2000) official method 923.03. Initially, the weight of clean and dried crucible was measured (W_1_) and a 5 g of sample (W_2_) was added and charred in the hot plate under the hood. The charred sample was placed in a muffle furnace (CARBOLITE, S336RB, England) and ignited at 550 °C for 5 h until the sample became white/gray. The crucibles and their content were cooled in a desiccator and weighed (W_3_) to determine ash content using Eq. (6).

(6) $$\text{Ash}\;\left(\text{\%}\right)=\frac{{\text{W}}_{3}-{\text{W}}_{1}}{{\text{W}}_{2}}\times 100$$

where; W_1_ - the weight of the crucible, W_2_ - the weight of the sample, and W_3_ - the weight of the crucible and sample after ashing.

#### 2.3.6 Total carbohydrate

Total carbohydrates were determined using Eq. (7) (Protein, fat, ash, fiber, and moisture proportions were added and subtracted from 100)

(7) Total Carbohydrate(%)=100−% of (Crude protein+Moisture+Ash+Crude fat+Crude fiber)

#### 2.3.7 Energy value

The gross energy value (expressed in kilocalories) was calculated using Atwater's conversion factors of 4 kcal/g for protein, 9 kcal/g for fat, 4 kcal/g for carbohydrates, and 2 kcal/g for fiber (FAO, 2003) (Eq. (8)).

(8) Gross energy(kcal/100 g)=((9×Crude fat)+(4×Crude protein)+(4×Total carbohydrate)+(2×Crude fiber))(8)

### 2.4 Determination of mineral content

Calcium (Ca) and magnesium (Mg) contents were measured using EDTA titration. The analysis of iron, zinc, and copper was done based on the Jorhem et al. (2019) method. Two grams of dry flour sample was charred over a hotplate until smoking ceases and then incinerated in a muffle furnace (Nabertherm, D-6072 Dreieich, Germany) at 550 °C for 3 h. The resulting white ash was weighed and dissolved in 3 ml of concentrated nitric acid and diluted up to 25 ml with deionized water. The standard stock solutions of Zn, Cu, and Fe were prepared using AAS grade standards. Atomic Absorption Spectrophotometry (SHIMADZU, AA-6880F, Japan) was used to determine the minerals Zn, Cu, and Fe using air/acetylene flame at wavelengths of 213.9, 324.8, and 248.3 nm, respectively. Different electrode lamps were used for each mineral. The equipment was run for standard solutions of each mineral before and during determination to check that it was working properly. To assess possible contamination, blank solutions were prepared containing the same reagents and using the same procedure as the samples and standards. Potassium content was determined by using Atomic Emission Spectrometry (AES) at 766.5nm wavelengths. Finally, Eq. (9) was used to calculate the element concentrations in the samples.

(9) $$\text{Metal content}\;\left(\text{mg}/100\text{ g}\right)=\frac{\left({\text{C}}_{\text{s}}-{\text{C}}_{\text{b}}\right)\times \text{V}}{\left(10\times \text{W}\right)}$$

where; C_s_ - the concentration of the sample in ppm (mg/L), C_b_ - the concentration of the blank in ppm (mg/L), V - the volume (ml) of the extract, W - the weight (g) of the samples.

### 2.5 Determination of anti-nutritional factors

#### 2.5.1 Phytate content

The phytate content was determined using the Latta and Eskin (1980) method. In brief, 0.1 g of sample was extracted at room temperature for 60 min with 10 ml 2.4 % HCl using a mechanical shaker, and the solution was centrifuged at 3000 rpm for 30 min. Two ml of wade reagent (0.3 % sulfosalicylic acid and 0.03 % FeCl_3_.6H_2_O in water) was added to 3 ml of the extract and thoroughly mixed with a Vortex for 5 s. A series of standard solutions of phytic acid was prepared in 0.2 N HCl, while de-ionized water was served as a blank. Both the standard and sample solutions (0.0–100 ppm and R^2^ = 0.996) were measured at 500 nm by using a UV-Vis spectrophotometer.

#### 2.5.2 Tannin content

Tannin was determined using the method described by (Burns, 1971). Two grams of sample flour were extracted for 24 h at room temperature using a mechanical shaker and 10 ml of methanol containing 1 % HCl. After 5 min of centrifugation at 1000 rpm, 1 ml of supernatant was taken and mixed with 5 ml of vanillin-HCl reagent (equal volumes of 8 % HCl in methanol and 4 % vanillin in methanol). Using D-catechin as a standard, the absorbance of the standard (0.0–1.4 mg/ml and R^2^ = 0.995) and sample solutions was measured after 20 min at 500 nm by using a UV-Vis spectrophotometer.

#### 2.5.3 Oxalate content

Extraction of total oxalates was carried out using the method as described in (Savage et al., 2000). A 40 ml 2.0 M HCL was shacked thoroughly with a 1.0 g freeze-dried powdered sample at 21 °C for 15 min to extract total oxalates. Oxalic acid content was determined using a High-Performance Liquid Chromatographic (HPLC) based on Castellari et al. (2000) methods. Before placing the frozen extract into 2 ml autosampler vials, it was thawed, homogenized, and filtered through a 0.45-m nylon syringe filter (Pall Corporation, Port Washington, NY, USA). The samples were analyzed using a Perkin-Elmer Series 200 HPLC system with 20 μl injections and a UV/VIS detector (Perkin-Elmer Corporation, Norwalk, CT, USA); absorbance was measured at 215 nm. Perkin-Elmer TotalChrom software (Version 6.2.1) was used to integrate the data. The sample concentration was estimated using a calibration curve (0–900 ppm and R^2^ = 0.999) based on the elution profiles of the oxalic acid standard.

### 2.6 Estimation of minerals bioavailability

Molar ratios of anti-nutrients to minerals predict the bioavailability of the minerals. The molar ratio between anti-nutrients and minerals was determined using the method described in (Hailu and Addis, 2016). The molar ratios of phytates: calcium, oxalates: calcium, phytates: iron, phytates: zinc, and phytates∗calcium: zinc and the critical values suggested were used to predict zinc, calcium, and iron bioavailability.

### 2.7 Experimental design and statistical analysis

A completely randomized design in three replicates was used to conduct the study. All statistical analyses were carried out using SPSS version 20.0 (SPSS Institute Inc., Cary, NC). Data were evaluated using a one-way analysis of variance (ANOVA). Duncan's multiple range test was used to identify significant differences among means. The result was reported as mean ± SE (standard error) and significance was declared at p < 0.05.

## 3 Results and discussion

### 3.1 Proximate composition

Table 1 shows the proximate compositions of the five oat varieties studied. Moisture, crude protein, crude fat, total ash, and gross energy contents showed significant (p < 0.05) differences among the varieties. However, crude fiber and total carbohydrate did not show significant differences (p > 0.05).

**Table 1:** Proximate composition (g/100 g, DWB)∗ of landrace and improved varieties of oat grains.

Oat Varieties	Moisture	Crude protein	Crude fat	Crude fiber	Total ash	Total carbohydrate	Gross energy (kcal/100 g)
Yellow	9.0 ± 0.14^ab^	15.0 ± 0.81^ab^	8.3 ± 0.01^b^	2.1 ± 0.15^a^	1.3 ± 0.00^a^	73.3 ± 0.66^a^	432.2 ± 0.29^b^
White	9.8 ± 0.38^a^	13.7 ± 0.02^bc^	8.6 ± 0.02^b^	2.3 ± 0.50^a^	1.2 ± 0.00^d^	74.3 ± 0.52^a^	433.8 ± 0.88^b^
Black	8.5 ± 0.04^b^	11.9 ± 0.05^c^	10.3 ± 0.46^a^	3.5 ± 0.44^a^	1.3 ± 0.00^a^	73.0 ± 0.70^a^	439.1 ± 0.62^a^
Goslin	8.9 ± 0.14^ab^	15.8 ± 0.53^a^	6.7 ± 0.01^c^	3.00 ± 0.37^a^	1.2 ± 0.00^c^	73.3 ± 0.17^a^	422.9 ± 0.72^c^
Soresi	9.2 ± 0.20^ab^	15.5 ± 0.05^ab^	8.2 ± 0.01^b^	2.5 ± 0.15^a^	1.2 ± 0.00^b^	72.6 ± 0.12^a^	430.8 ± 0.21^b^
**Mean**	**9.1 ± 0.14**	**14.4 ± 0.41**	**8.4 ± 0.030**	**2.7 ± 0.19**	**1.2 ± 0.02**	**73.3 ± 0.24**	**431.8 ± 1.42**
**CV**	**5.9**	**11.1**	**14**	**27.6**	**5.5**	**1.3**	**1.3**

Data are expressed as mean ± standard error of replicate (n = 3). Means that do not share the same letter down the column are significantly different.

The moisture content ranged from 8.5 to 9.8 % for all studied varieties. All varieties except black landrace showed no significant difference in terms of moisture content. Moisture content value below 10 % ensures the safe storage of the grains. The lower value was obtained for the black landrace variety (8.5 g/100 g) and the higher value was obtained for the white landrace (9.8 g/100 g). The average moisture content was 9.1 g/100 g, which is similar to the moisture content of oats varieties reported in different works (6.7–10.8 %) (Youssef et al., 2016; Amanuel et al., 2019).

The crude protein content also showed a significant difference (p < 0.05), the higher content was obtained for Goslin (15.8 g/100 g) and the lowest was obtained for black-colored oat (11.9 g/100 g) on a dry matter (DM) basis. Our result is in close agreement with the crude protein content of oats studied by different authors. Crude protein contents of 10.9–16.6 g/100 g were reported in different works (Mosissa et al., 2018; Sterna et al., 2016; Kudake et al., 2017; Van den Broeck et al., 2015). However, values are slightly higher than those reported by Chappell et al. (2017) (9.18 g/100 g) and the minor difference could be related to the genetic differences among oat types studied and agronomic practices applied during production. For instance, Chappell et al. (2017) found a strong correlation of rainfall with the protein content of oat grains. In general, the crude protein content of the landraces commonly used in a specific location of Gozamin districts consists of comparable value to other oat varieties grown and used elsewhere in the world.

However, as compared to other cereals, oat grain has a relatively higher protein content. Ahmed et al. (2014) indicated that the crude protein content of wheat, rice, and maize were 9.1, 7.2, and 9.1 g/100 g, respectively, which are less than the findings of this study. This implies that the oats used in this study could have good potential to contribute as a low-cost protein source in regions where protein-energy malnutrition is a problem.

The crude fat contents also differed significantly (p < 0.05) among studied varieties (6.7–10.3 g/100 g). The highest value was obtained for black landrace (10.3 g/100 g) and the lowest was for Goslin, an improved variety (6.7 g/100 g). The remaining three varieties showed no significant differences (p > 0.05) in their crude fat content. Results showed that the landraces had higher crude fat contents than the improved varieties.

This study's findings are in line with what was reported by different authors (Kudake et al., 2017; McKevith, 2004; Butt et al., 2008). However, the results were slightly higher than the reported values of Chappell et al. (2017) (6.5 g/100 g), Sandhu et al. (2017) (4.2–5.3 g/100 g), and Sangwan et al. (2014) (4.5 g/100 g). In comparison to other cereals, this study's crude fat values are higher than the commonly consumed cereals in Ethiopia such as wheat (1.7 g/100 g) (Ahmed et al., 2014), barley (2.7 g/100 g) (Chappell et al., 2017), maize (3.5 g/100 g) (Ahmed et al., 2014), teff (2.5 g/100 g) (Baye, 2014), rice (0.4 g/100 g) (Ahmed et al., 2014), sorghum (3.7–3.9 g/100 g) (Durojaiye et al., 2016), and rye (2 g/100 g) (McKevith, 2004). As a potential source of crude fats, landrace and improved varieties are likely to have a higher potential for essential fatty acid content than other commonly consumed cereals. They could also serve as a carrier for fat-soluble vitamins (Zhou et al., 1998).

The ash content of the food sample has a positive correlation with the mineral content of the grains. The ash content differed significantly (p < 0.05) between landrace and improved varieties, with values ranging from 1.2 g/100 g–1.3 g/100 g. The maximum value was found for yellow and black-colored oats, while the minimum value was found for white-colored oats. The mean ash content of oats in this study (1.2 g/100 g) was lower than that reported by Chappell et al. (2017) and Kudake et al. (2017) (1.8 g/100 g), and Sandhu et al. (2017) and Sangwan et al. (2014) (2.7–3.5 g/100 g). However, the variation in total ash content might also be influenced by dehulling practice, genetic variation, or implemented agronomic practices. Compared to other grains, the total ash content was similar to that of barley (1.3 g/100 g), maize (1.2 g/100 g), sorghum (1.3 g/100 g), and less than wheat (1.6 g/100 g), millet (2.4 g/100 g), and teff (3.8 g/100 g (EHNRI, 1998).

The crude fiber and total carbohydrate contents showed no significant difference (p > 0.05) among studied oat varieties. The crude fiber contents ranged from 2.1 g/100 g (yellow-colored variety) to 3.5 g/100 g (black-colored variety). The mean crude fiber content (2.7 g/100 g) was comparable to the results of previous works, values ranged from 2.1 - 4.2 g/100 g were reported by (Kudake et al., 2017; Biel et al., 2014). Slightly higher crude fiber contents, 3.5–5.9 g/100 g, were reported by (Youssef et al., 2016). However, results in this study are relatively higher than the fiber content of rice (0.6–1.0 g/100 g), sorghum (0.6 g/100 g), and wheat (2 g/100 g) (Baye, 2014). Epidemiological evidence suggests that a high fiber diet may help to reduce the occurrence of certain chronic non-communicable diseases like coronary heart disease, diabetes, colon cancer, obesity, high blood pressure, and various gastrointestinal problems (Pinto-Sánchez et al., 2017; Rasane et al., 2013).

Total carbohydrate in oats ranged from 72.6 to 74.3 g/100 g. A slightly lower result for oats (64.7 g/100 g) was reported by (Kudake et al., 2017). Like other cereals, the total carbohydrate of oats in this study makes up the largest major nutrient proportion. However, compared to other cereals, the results of this study are comparable to the majority of grains consumed in Ethiopia (EHNRI, 1998). The total carbohydrate contents for barley, maize, wheat, millet, rice, sorghum, and teff were 78.8 g/100 g, 76 g/100 g, 76.6 g/100 g, 78.5 g/100 g, 81.5 g/100 g, 80.4 g/100 g, 73.1 g/100 g, respectively (EHNRI, 1998).

The gross energy contents among oat varieties ranged from 422.9 kcal/100 g to 439.1 kcal/100 g. As depicted in Table 1, the black-colored oat had significantly higher (p < 0.05) gross energy content (439.1 kcal/100 g) followed by white-colored oat (433.8 kcal/100 g), yellow-colored oat (432.2 kcal/100 g), Soresi variety (430.8 kcal/100 g), and Goslin (422.9 kcal/100 g). In this study, the gross energy contents of oats were found to be high when compared to the most commonly consumed cereals in Ethiopia. According to the Ethiopian food composition table, the gross energy values (kcal/100 g) of barley, maize, wheat, millet, rice, sorghum, and teff were 370.9, 376, 379.7, 350.4, 357.2, 377.4, and 355.1, respectively (EHNRI, 1998). Baye (2014) also reported the gross energy values of commonly consumed cereals in Ethiopia, which were lower than those indicated in this study. The higher gross energy values of oats could be attributed to their remarkably higher crude fat content that contributes a great share to their gross energy values.

### 3.2 Mineral contents

Table 2 presents the mineral contents of the oat varieties. Iron, copper, zinc, potassium, calcium, and magnesium showed significant (p < 0.05) variation among the oat varieties. Minerals are inorganic elements that have essential metabolic functions which cannot be produced by living organisms. Trace minerals are less than 1 % of the minerals in our body but are essential for our life (Gordon and Hampl, 2007). Minerals play a significant role in structural functions involving soft tissues and the skeleton, as well as regulatory functions such as blood clotting, oxygen transport, neuromuscular transmission, and enzymatic activity (NRC, 1989). Most of the physiological functions of minerals are closely related to their role in enzyme activity.

**Table 2:** Trace and major minerals content (mg/100 g, DWB)∗ of landrace and improved varieties of oat grains.

Oat Varieties	Trace minerals			Major minerals		
	Iron	Copper	Zinc	Potassium	Calcium	Magnesium
Yellow	3.0 ± 0.06^a^	0.4 ± 0.01^a^	2.0 ± 0.01^b^	258.3 ± 0.54^a^	44.0 ± 0.41^d^	89.1 ± 0.17^a^
White	2.8 ± 0.00^b^	0.3 ± 0.01^c^	1.8 ± 0.00^c^	250.0 ± 0.07^b^	44.0 ± 0.09^d^	80.2 ± 0.03^b^
Black	3.0 ± 0.00^a^	0.2 ± 0.00^d^	1.6 ± 0.01^d^	250.0 ± 0.50^b^	58.7 ± 0.01^c^	80.2 ± 0.02^b^
Goslin	2.5 ± 0.00^bc^	0.4 ± 0.01^b^	2.0 ± 0.00^b^	241.7 ± 0.19^c^	102.7 ± 0.39^a^	62.4 ± 0.21^c^
Soresi	2.6 ± 0.01^bc^	0.3 ± 0.01^c^	2.0 ± 0.01^a^	241.7 ± 0.42^c^	73.3 ± 0.02^b^	80.2 ± 0.01^b^
**Mean**	**2.8 ± 0.05**	**0.3 ± 0.06**	**1.88 ± 0.04**	**248.3 ± 1.67**	**64.5 ± 5.67**	**78.4 ± 2.33**
**CV**	**7.4**	**21.8**	**8.3**	**2.8**	**38**	**12.5**

Data are expressed as mean ± standard error of replicate determinations (n = 3). Means that do not share the same letter down the column are significantly different.

#### 3.2.1 Trace minerals (Fe, Cu, Zn)

As indicated in Table 2, iron content differed significantly (p < 0.05) among the oat varieties and ranged from 2.5 to 3.0 mg/100 g. Oat with yellow color was the highest in its iron content, and Goslin was the lowest. The iron contents of yellow (3 mg/100 g) and black (3 mg/100 g) colored oats were higher than other varieties but showed no significant difference (p > 0.05) between them. Goslin and Soresi improved varieties exhibited lower iron contents of 2.5 and 2.6 mg/100 g, respectively, as compared to the landraces, but they did not significantly differ (p > 0.05) from each other (Table 2).

The mean iron content (2.8 mg/100 g) of the oat varieties was lower than what was reported in different literature (Chappell et al., 2017; Youssef et al., 2016). Özcan et al. (2017) also reported the iron contents of many different oat varieties, ranging from 3.0 to 8.1 mg/100 g. However, the obtained results were higher than the iron content of rice (0.4 mg/100 g) and comparable to maize (2.4 mg/100 g) (Meherunnahar et al., 2018). McKevith (2004) also reported the iron contents of wheat (2 mg/100 g), rice (1.4 mg/100 g), corn (1.1 mg/100 g), which were also lower than the iron contents of oats in this study.

The copper content was significantly different (p < 0.05) among oats varieties, and the value ranged between 0.2 to 0.4 mg/100 g. Yellow-colored and Goslin varieties had highest copper content (0.4 mg/100 g) followed by white-colored (0.3 mg/100 g) and black-colored (0.2 mg/100 g) ones (Table 2). However, no significant difference (p > 0.05) was observed between Soresi (0.3 mg/100 g) and white-colored oat.

Copper is a component of several oxidoreductase enzymes, which act as a co-factor of antioxidant enzymes to protect the human body from radicals associated with oxidative stress. It also stimulates the immune system, which aids in the fight against infections, tissue repair, and healing. It is also essential to form hemoglobin and keeps bones, blood vessels, and nerves healthy (Berdanier et al., 2002). This study's mean value (0.3 mg/100 g) is lower than the reported values of oat by Sangwan et al. (2014) (0.6 mg/100 g). Anderson et al. (2012) reported the copper contents of wheat (0.6 mg/100 g), barley (0.5 mg/100 g), and sorghum (0.5 mg/100 g), which were slightly higher than the copper contents of oats in this study. This suggests that the oats used in this study are high in copper.

The zinc content of oats ranged from 1.6 to 2.1 mg/100 g. Similar to other trace mineral elements, landraces and improved varieties showed significant differences (p < 0.05) in their zinc content. As compared to landraces, improved varieties exhibited better zinc content (Table 2). Soresi scored the highest zinc content, and black-colored oat recorded the lowest. Özcan et al. (2017) indicated that zinc contents of oats ranged between 1.5 mg/100 g and 3.8 mg/100 g, which is in close agreement with our result. However, our finding is slightly lower than the values reported by Sangwan et al. (2014) (3.3–4.5 mg/100 g). The variation could be related to a genetic difference and agronomic practices applied during production. McKevith (2004) reported zinc contents of rice (1.8 mg/100 g), corn (1.7 mg/100 g), and barley (2.1 mg/100 g), which were comparable with the zinc contents of oats in this study. Zinc is desirable for the proper growth and maintenance of the human body. It is a vital component of a large number (>300) of enzymes participating in the synthesis and degradation of carbohydrates, lipids, proteins, and nucleic acids (WHO, 2005). It is found in several systems and biological reactions, and it is also needed for immune function, wound healing, blood clotting, and thyroid function. Therefore both the landrace and improved varieties of oats can be considered as a potential source of zinc.

#### 3.2.2 Major minerals (K, Ca, Mg)

Table 2 shows the major minerals determined from different landraces and improved varieties of oat. The potassium content varied from 241.7 to 258.3 mg/100 g and significantly differed among oat varieties. Landraces were better in potassium content (yellow > white = black) as compared to improved varieties (Table 2). The average potassium content (248.3 mg/100 g) is lower than what was reported by Özcan et al. (2017) (305–562 mg/100 g). McKevith (2004) reported the potassium contents of wheat (150 mg/100 g), rice (250 mg/100 g), maize (220 mg/100 g), and barley (270 mg/100 g), which were comparable with the potassium contents of oats in this study. Potassium is a necessary nutrient for maintaining total body fluid, electrolyte balance, and cellular function. WHO recommends that adults consume at least 3510 mg of potassium per day (WHO, 2012). Thus, daily consumption of 100 g oat food could meet 7 % of the requirement. A high potassium accumulation in the body promotes iron utilization which is useful for people who take diuretics to regulate hypertension (Berdanier et al., 2002). Under hot weather and strenuous physical activity, outflows of potassium in sweat are boosted; however, acclimation occurs quickly, and potassium losses through sweat are reduced. As a result, most people can get enough potassium from their diet without needing supplements or products that have been specially formulated (WHO, 2012). According to the findings of this study, oats are a potential grain source of potassium.

Calcium is used to build bones and teeth and is involved in the function of the muscular system. Over 99 % of total body calcium is found in teeth and bones (Berdanier et al., 2002). The calcium contents of the oats ranged from 44 to 102.7 mg/100 g. Goslin variety exceptionally (p < 0.05) high in calcium (102.7 mg/100 g) content compared to others. The improved varieties, Goslin and Soresi (73.3 mg/100 g), had higher calcium contents than the landrace varieties. The mean calcium content of the oats was 64.5 mg/100 g. It was consistent with the results of Youssef et al. (2016), who reported 54–71 mg/100 g, but higher than the values reported by Sangwan et al. (2014) (53.9 mg/100 g) and Chappell et al. (2017) (54.9 mg/100 g). On the other hand, higher calcium content that ranged between 56.9 and 127 mg/100 g was also reported by Özcan et al. (2017), which could be associated with different factors such as growth conditions, genetic factors, geographical variations, and analytical procedures used for determination. According to the Ethiopian food composition table, the calcium content (mg/100 g) of barley, corn, wheat, rice, sorghum, and teff are 28, 16, 12, 12, 9, and 1.2, respectively, by far lower than calcium content of oats in this study (EHNRI, 1998).

The magnesium contents of oats varied from 62.4 to 89.1 mg/100 g. Yellow oat had significantly higher (p < 0.05) magnesium content, and it was followed by white-colored, black-colored, and Soresi oats. Unlike its highest calcium content, Goslin's magnesium content was the lowest. The mean concentration of magnesium (78.4 mg/100 g) in this study was lower than the contents reported by many authors. The content of magnesium reported by Youssef et al. (2016) was 112–120 mg/100 g and Özcan et al. (2017) was 202.5–225.3 mg/100 g Jakobsone et al. (2019) studied the macro and trace elements in oat cultivars bred in Latvia and they found that weather conditions (air temperature and rainfall) influence the amounts of calcium, potassium, sodium, and magnesium.

According to the reports of McKevith (2004), the magnesium contents of maize (81 mg/100 g) and barley (65 mg/100 g) were comparable. While the magnesium contents of wheat (20 mg/100 g) and rice (32 mg/100 g) were lower than our result.

Magnesium is the body's fourth most abundant cation, following potassium in its intracellular concentration. This concentration reflects that magnesium is critical for many cellular functions, including oxidative phosphorylation, glycolysis, DNA transcription, fatty acid degradation, and protein synthesis (Monga et al., 2015; Berdanier et al., 2002). Magnesium, which forms a soluble complex with oxalate, reduces the chance of calcium oxalate forming kidney stones (Monga et al., 2015). This study suggests that sufficient magnesium can be obtained from consuming cereals like oats.

### 3.3 Anti-nutritional factors

Table 3 shows the anti-nutritional contents of the oat varieties. Phytate, tannin, and oxalate contents showed significant (p < 0.05) differences among the oat varieties.

**Table 3:** Phytate, tannin, and oxalate contents (mg/100 g, DWB)∗ of landrace and improved varieties of oat grains.

Oat Varieties	Phytates	Tannins	Oxalates
Yellow	269.6 ± 0.52^d^	46.1 ± 0.13^b^	71.4 ± 0.29^a^
White	276.2 ± 0.19^c^	51.5 ± 0.31^a^	50.6 ± 0.20^b^
Black	293.0 ± 0.39^a^	45.9 ± 0.84^b^	28.2 ± 0.16^d^
Goslin	272.1 ± 0.98^d^	41.1 ± 0.29^c^	51.7 ± 0.23^b^
Soresi	282.6 ± 0.93^b^	38.8 ± 0.13^d^	40.0 ± 0.41^c^
**Mean**	**278.7 ± 2.27**	**44.7 ± 1.19**	**48.4 ± 3.83**
**CV**	**3.4**	**11.1**	**33.1**

Data are expressed as mean ± standard error of replicates (n = 3). Means that do not share the same letter down the column are significantly different.

Anti-nutritional factors reduce the overall absorption of nutrients, particularly minerals, proteins, and vitamins, as a result, optimal nutrient utilization is hampered, and nutritive values are reduced (Whitney and Rolfes, 2011). Therefore, the lower the anti-nutrient factor in a given food is the better from a health point of view (Bora, 2014).

Phytate content showed a significant (p < 0.05) difference and the value ranged from 269.6 (yellow-colored) to 293.0 mg/100 g (black-colored). The landraces (yellow and white-colored oats) contain a relatively lower phytate content compared with improved variety Soresi.

In this study, the average phytate content of oats was lower (278.7 mg/100 g) than the value obtained by Norhaizan and Ain (2009) (394.9 mg/100 g). Samtiya et al. (2020) reported that the phytate contents of maize, rice, pearl millet, and wheat were 87.2–683.2 mg/100 g, 93.70 mg/100 g, 5.00 mg/100 g, and 795–800 mg/100 g, respectively. Olukemi et al. (2016) also showed that the phytate contents (mg/100 g) of wheat, maize, and rice were 121.8, 82.8, and 27.2, respectively.

Tannin forms a strong complex with protein and other macromolecules Berdanier et al. (2002), thus decreasing digestibility (McKevith, 2004). It also adversely influences the bioavailability of non-heme iron, resulting in low iron and calcium absorption. The tannin content significantly differed among oat varieties and ranged from 38.8 to 51.5 mg/100 g. Unlike phytate content, the tannin content of landraces is higher than that of improved varieties. The white-colored ones contain the highest value, followed by black and yellow-colored ones.

Tannic acid has a total acceptable daily intake of 560 mg for adult men and women (Sandberg, 2002). The obtained tannin content of the oats was lower than the acceptable daily intake level, assuming 100 g of oat foods was consumed per day. Processing treatments, like the germination of grains, dough fermentation, and boiling or baking, will further reduce the concentration.

Oxalate contents ranged from 28.16 mg/100 g for black-colored oat to 71.4 mg/100 g for yellowish oat. The oxalate content of oats was comparable to wheat. According to Siener et al. (2006), the oxalate contents of wheat were 53.3–76.6 mg/100 g.

Oxalate is a metabolic end-product of ascorbate, glyoxylate, and glycine metabolism in humans. It forms water-soluble salts with Na^+^, K^+^, and NH_4_^+^ ions; it also binds with Ca^2+^, Fe^2+^, and Mg^2+^ rendering these minerals unavailable to the cells (Kumar et al., 2017). Patients are currently advised to consume no more than 40–50 mg of oxalate per day (Brzezicha-Cirocka et al., 2015). Except for the yellow variety (71.4 mg/100 g), the oxalate content of all oats included in this study was lower than the acceptable daily intake levels assuming 100 g of oat foods was consumed per day.

### 3.4 Mineral bioavailability and molar ratios

Table 4 shows the molar ratios of anti-nutrients to minerals. Phytates: calcium, oxalates: calcium, phytates: iron, phytates: zinc, and phytates∗calcium: zinc showed significant variation (p < 0.05) among oat varieties.

**Table 4:** Calculated molar ratios of anti-nutrients (phytates, oxalates) to minerals (Ca, Fe, Zn) of oat grains.

Oat Varieties	(Phytates: Ca)^1^	(Oxalates: Ca)^2^	(Phytates: Fe)^3^	(Phytates: Zn)^4^	(Phytates∗Ca: Zn)^5^
Yellow	0.37 ± 0.00^a^	0.74 ± 0.00^a^	7.60 ± 0.15^c^	13.73 ± 0.10^c^	15.08 ± 0.25^d^
White	0.38 ± 0.00^a^	0.52 ± 0.00^b^	8.49 ± 0.03^b^	15.06 ± 0.08^b^	16.53 ± 0.13^c^
Black	0.30 ± 0.00^b^	0.22 ± 0.00^d^	8.35 ± 0.03^b^	17.66 ± 0.10^a^	25.86 ± 0.15^b^
Goslin	0.16 ± 0.00^d^	0.23 ± 0.00^d^	9.07 ± 0.02^a^	13.68 ± 0.07^c^	35.05 ± 0.21^a^
Soresi	0.23 ± 0.00^c^	0.25 ± 0.00^c^	9.05 ± 0.05^a^	13.79 ± 0.08^c^	25.23 ± 0.14^b^
**Mean**	**0.29 ± 0.02**	**0.39 ± 0.05**	**8.51 ± 0.15**	**14.78 ± 0.41**	**23.54 ± 0.93**
**CV**	**32.5**	**59**	**7.1**	**11.6**	**34.3**

Means not followed by the same superscript letters in each column of the oats are significantly different (p < 0.05) from each other. **Notes**: ^1^mg of phytates/molecular weight of phytates: mg of calcium/molecular weight of calcium; ^**2**^mg of oxalates/molecular weight of oxalate: mg of calcium/molecular weight of calcium; ^**3**^mg of phytates/molecular weight of phytates: mg of iron/molecular weight of iron; ^**4**^mg of phytates/molecular weight of phytates: mg of zinc/molecular weight of zinc; ^**5**^(mg of calcium/molecular weight of calcium)∗(mg of phytates/molecular weight of phytates)/(mg of zinc/molecular weight of zinc).

The fraction of an element that is solubilized and finally absorbed from the gastrointestinal tract into the systemic circulation of humans and animals is referred to as bioavailability (Endraiyani, 2008). Only some of the minerals in the food will be absorbed in the gastrointestinal tract due to the presence of dietary fiber, phytates, tannins, and oxalates (Noonan and Savage, 1999). Minerals with low bioavailability cause health imbalances and impaired vital functions, such as anemia and osteoporosis, which are common in both developed and developing countries (Norhaizan and Ain, 2009).

Oat phytate: Ca molar ratios ranged from 0.16 to 0.38 (Table 4). The maximum value belongs to the white oat, and the minimum value belongs to the Goslin variety. The molar ratio of phytate: Ca, which is less than 0.24, indicates adequate calcium bioavailability in food (Ma et al., 2007). The molar ratios of phytate: Ca of oat varieties such as yellow, black, and white-colored oats were less than the earlier described critical molar ratios, indicating that calcium absorption was not adversely affected, however, Goslin and Soresi varieties were above the critical value, exhibiting a potential inhibitory effect of phytates on the calcium bioavailability.

Castro-Alba et al. (2019) studied the mineral bioavailability of Bolivian foods and reported that the ratio of phytate: Ca of oat was four. They also reported a relatively higher ratio for corn (12.2) and rice (10.36) but a relatively lower ratio for wheat (3.07) and barley (0.61).

The oxalate: Ca molar ratios of the oats ranged from 0.22 (black-colored oat) to 0.74 (yellowish oat). The effect of oxalate content on total dietary calcium bioavailability is only significant when the oxalate: Ca ratio is higher than one (Frontela et al., 2009). According to this study, the molar ratios of oat varieties were less than the limiting ratio of oxalate: Ca, which suggests that oxalates may not have a negative impact on dietary calcium bioavailability in the oat varieties studied.

The phytate: Fe molar ratios ranged from 7.6 to 9.1 mg/100 g. The Goslin variety had the highest value, while the yellowish oat had the lowest value. The mean phytates: Fe molar ratio in this study was 8.51, which is comparable to the reports of Norhaizan and Ain (2009) for oats (9.34). The same authors also reported comparable phytates: Fe molar ratio with wheat (8.06) and relatively lower ratios for rice (3.26).

When phytates: Fe molar ratios fall below one, their inhibition on iron absorption begins to fade; however, ratios as small as 0.2 have some effect (Ma et al., 2007).

The current results indicated that the phytates: Fe molar ratios of oats were greater than the critical value, implying that phytates have a significant impact on iron bioavailability. According to Norhaizan and Ain (2009), iron bioavailability can be increased by adding fruits rich in vitamin C in breakfast cereals or by having fish, eggs, and chicken in daily meals, which will help increase iron absorption from our meal. The ratios of phytates: Fe in this research are much lower than those reported by Castro-Alba et al. (2019) (49.2). Compared to other cereals, the outcome of this finding is higher than that described for barley (2.6) and lower than that of corn (15.1) (Castro-Alba et al., 2019).

The quality of food as a source of zinc depends on the amount of zinc present as well as the amount of other dietary constituents (Endraiyani, 2008). By forming undissolved mineral chelates at physiological pH, phytates reduce dietary zinc bioavailability. The relative zinc content and phytic acid affect the formation of chelates. The molar ratio of phytate: Zn is thought to be a strong predictor of zinc bioavailability when compared to total phytate. The molar ratio of phytate: Zn among the oat varieties ranged from 13.68 (Goslin variety) to 17.66 (black-colored oat). The mean phytate: Zn molar ratio in this study was 14.78, which was comparable with the results of Norhaizan and Ain (2009) (13.29). Foods with a phytate: Zn molar ratio less than 10 are thought to have sufficient zinc availability, and the value greater than 15 showed insufficient availability (Gemede, 2020). Except for the white (15.1) and black (17.7) colored oats, the phytate: Zn molar ratios of the other varieties were less than the limiting molar ratio. The ratio of phytate: Zn in this study is much lower than the molar ratio phytate: Zn of oat reported by (Castro-Alba et al., 2019) (82.4). In comparison to other cereals, the outcome of this study is less than that of barley (24) and comparable to that of yellow corn (14.6) (Castro-Alba et al., 2019).

Because calcium has a substantial impact on the absorption of zinc in the cases of excess phytate intakes, it has been proposed that the [phytates][Ca]/[Zn] molar ratios may be a more reliable predictor for the bioavailability of zinc than the phytate: Zn molar ratio alone (WHO, 1996). Significant variations were observed in the [phytates][Ca]/[Zn] molar ratios of the oat varieties. It ranged from 15.1 (yellow-colored) to 35.1 (Goslin variety). High levels of calcium in foods can facilitate the decrease in zinc estimated bioavailability induced by phytates when the [phytates][Ca]/[Zn] molar ratio exceeds 0.5 mol/kg (Adetuyi and Osagie, 2011). In this study, the values recorded for all oats varieties were higher than the limiting molar ratio, which implies the high impact of phytates on bioavailability and zinc absorption. The [phytates][Ca]/[Zn] ratios in this study are much lower than that reported for oats (820) barley (140) and corn (18.4) (Castro-Alba et al., 2019).

To decrease the inhibitory effect of anti-nutrients on minerals, food processing and fortification with micronutrients and the use of nutrient absorption enhancers have been recommended. Soaking whole grains, dehulling, malting, and fermentation are some of the processing methods for reducing anti-nutrient compounds. Fortification with micronutrients is also another option to increase the bioavailability of nutrients (Norhaizan and Ain, 2009).

## 4 Conclusions

Noticeable differences in the proximate, minerals, and anti-nutrient contents of the different landraces and improved oat varieties cultivated in Ethiopia were observed. The proximate values of oats are similar to other varieties available elsewhere, and most of the studied nutritional parameters are better than commonly consumed staple cereals in Ethiopia. The bioavailability of minerals, except for iron, was adequate for landrace and improved varieties considered in this study. They have shown an excellent source of valuable nutrients that can contribute significantly to the human diet and nutrition. Hence, these underutilized indigenous landraces and improved varieties need to be considered for further expansion and commercialization in other regions of the country or other developing countries to contribute for ensuring food and nutrition security.

## Declarations

### Author contribution statement

Getaneh Firew Alemayehu: Conceived and designed the experiments; Performed the experiments; Analyzed and interpreted the data; Contributed reagents, materials, analysis tools or data; Wrote the paper.

Sirawdink Fikreyesus Forsido, Yetenayet B. Tola, Endale Amare: Conceived and designed the experiments; Analyzed and interpreted the data; Wrote the paper.

Minbale Adimas Teshager, Addisu Alemayehu Assegie: Contributed reagents, materials, analysis tools or data.

### Funding statement

This research did not receive any specific grant from funding agencies in the public, commercial, or not-for-profit sectors.

### Data availability statement

The data that has been used is confidential.

### Declaration of interests statement

The authors declare no conflict of interest.

### Additional information

No additional information is available for this paper.

## References

[bib1] Adetuyi F., Osagie A. (2011). Nutrient, antinutrient, mineral and zinc bioavailability of okra Abelmoschus esculentus (L) Moench variety. Am. J. Food. Nutr..

[bib2] Ahmed K., Shoaib M., Akhtar M.N., Iqbal Z. (2014). Chemical analysis of different cereals to access nutritional components vital for human health. Int. J. Chem. Biochem. Sci..

[bib3] Amanuel W., Kassa S., Deribe G. (2019). Biomass yield and nutritional quality of different oat varieties (*Avena sativa*) grown under irrigation condition in Sodo Zuriya District, Wolaita Zone, Ethiopia. Agri. Res. Tech..

[bib4] Anderson V., Lardy G., Bauer M., Swanson K., Zwinger S. (2012). Barley Grain and Forage for Beef Cattle.

[bib5] AOAC (2000).

[bib6] Baye K. (2014). Teff: Nutrient Composition and Health Benefits.

[bib7] Berdanier C.D., Dwyer J.T., Heber D. (2002). Hand Book of Nutrition and Food.

[bib8] Biel W., Jacyno E., Kawęcka M. (2014). Chemical composition of hulled, dehulled and naked oat grains. S. Afr. J. Anim. Sci..

[bib9] Bora P. (2014). Antinutritional factors in foods and their effects. J. Acad. Ind. Res..

[bib10] Brzezicha-Cirocka J., Grembecka M., Szefer P. (2015). Oxalate, magnesium and calcium content in selected kinds of tea: impact on human health. Eur. Food Res. Technol..

[bib11] Burns R.E. (1971). Methods for estimation of tannin in grain sorghum. Agron. J..

[bib12] Butt M.S., Tahir-Nadeem M., Khan M.K.I., Shabir R., Butt M.S. (2008). Oat: unique among the cereals. Eur. J. Nutr..

[bib13] Castellari M., Versari A., Spinabelli U., Galassi S., Amati A. (2000). An improved HPLC method for the analysis of organic acids, carbohydrates, and alcohols in grape musts and wines. J. Liq. Chromatogr. Relat. Technol..

[bib14] Castro-Alba V., Lazarte C.E., Bergenståhl B., Granfeldt Y. (2019). Phytate, iron, zinc, and calcium content of common Bolivian foods and their estimated mineral bioavailability. Food Sci. Nutr..

[bib15] Chappell A., Scott K.P., Griffiths I.A., Cowan A.A., Hawes C., Wishart J., Martin P. (2017). The agronomic performance and nutritional content of oat and barley varieties grown in a northern maritime environment depends on variety and growing conditions. J. Cereal. Sci..

[bib16] Decker E.A., Rose D.J., Stewart D. (2014). Processing of oats and the impact of processing operations on nutrition and health benefits. Br. J. Nutr..

[bib17] Durojaiye I.A., Drambi U.D., Chukwu O. (2016). An evaluation of proximate composition on cereal grains for confectionery and pasta production. Int. Refereed J. Eng. Sci..

[bib18] EHNRI (1998). Food Composition Table for Use in Ethiopia.

[bib19] Endraiyani V. (2008). Iron (Fe) Bioavailability from Mung Beans: Effects of Household Processing and Form of Fe Fortificants.

[bib20] Fanzo J., Hunter D., Borelli T., Mattei F. (2013). Diversifying Food and Diets: Using Agricultural Biodiversity to Improve Nutrition and Health.

[bib21] FAO (2003). Food energy-methods of analysis and conversion factors. Report of a Technical Workshop, Rome: Food and Nutrition Paper 77.

[bib22] Frison E., Smith I., Johns T., Cherfas J., Eyzaguirre P. (2005). Using biodiversity for food, dietary diversity, better nutrition and health. S. Afr. J. Clin. Nutr..

[bib23] Frontela C., Scarino M.L., Ferruzza S., Ros G., Martínez C. (2009). Effect of dephytinization on bioavailability of iron, calcium and zinc from infant cereals assessed in the Caco-2 cell model. World J. Gastroenterol..

[bib24] Gemede H.F. (2020). Nutritional and antinutritional evaluation of complementary foods formulated from maize, pea, and anchote flours. Food Sci. Nutr..

[bib25] Getaneh F.A., Forsido S.F., Yetenayet B.T., Addisu A.A., Minbale A.T., Endale A. (2021). Traditional food processing practices of oats (*Avena sativa*) and its contribution to food security in Gozamin district of northwest Ethiopia. Afr. J. Food Agric. Nutr. Dev..

[bib26] Gordon M.W., Hampl J.S. (2007). Perspectives in Nutrition.

[bib27] Hailu A.A., Addis G. (2016). The content and bioavailability of mineral nutrients of selected wild and traditional edible plants as affected by household preparation methods practiced by local community in Benishangul Gumuz Regional State, Ethiopia. Int. J. Food Sci..

[bib28] Hareland G.A., Manthey F.A. (2003). Oats. Encyclopedia of Food Sciences and Nutrition.

[bib29] Jakobsone I., Zute S., Bleidere M., Kantane I., Ece L., Bartkevics V. (2019). Macro and trace elements in oat cultivars bred in Latvia. Zemdirb*.* Agric..

[bib30] Jorhem L., Afthan G., Cumont G., Dypdahl H.P., Gadd K., Havre G.N., Julshamn K., Kåverud K., Lind B. (2019). Determination of metals in foods by atomic absorption spectrometry after dry ashing: NMKL1 collaborative study. J. AOAC Int..

[bib31] Kudake D., Pawar V., Muley A.B., Parate A.R.V., Talib I.M. (2017). Enrichment of wheat flour noodles with oat flour: effect on physical, nutritional, antioxidant and sensory properties. Int. J. Curr. Microbiol. Appl. Sci..

[bib32] Kumar B., Tirkey N., Kumar S. (2017). Anti-nutrient in fodders: a review. Chem. Sci. Rev. Lett..

[bib33] Latta M., Eskin M. (1980). A simple and rapid colorimetric method for phytate determination. J. Agric. Food Chem..

[bib34] Ma G., Li Y., Jin Y., Zhai F., Kok F.J., Yang X. (2007). Phytate intake and molar ratios of phytate to zinc, iron and calcium in the diets of people in China. Eur. J. Clin. Nutr..

[bib35] Mäkinen O.E., Sozer N., Ercili-Cura D., Poutanen K. (2017). Protein from oat: structure, processes, functionality, and nutrition. Sustainable Protein Sources.

[bib36] McKevith B. (2004). Nutritional aspects of cereals. Nutr. Bull..

[bib37] Meherunnahar M., Chowdhury R., Hoque M., Satter M., Islam M. (2018). Comparison of nutritional and functional properties of BK2 foxtail millet with rice, wheat and maize flour. Progress. Agric..

[bib38] Monga M., Penniston K.L., Goldfarb D.S. (2015). Pocket Guide to Kidney Stone Prevention: Dietary and Medical Therapy.

[bib39] Mosissa F., Kefala B., Abeshu Y. (2018). Potential of oats (*Avena sativa*) for food grain production with its special feature of soil acidity tolerance and nutritional quality in central highlands of Ethiopia. Adv. Crop Sci. Tech..

[bib40] Nielsen S.S. (2010). Food Analysis.

[bib41] Noonan S., Savage G. (1999). Oxalate content of foods and its effect on humans. Asia Pac. J. Clin. Nutr..

[bib42] Norhaizan M., Ain A.N.F. (2009). Determination of phytate, iron, zinc, calcium contents and their molar ratios in commonly consumed raw and prepared food in Malaysia. Mal. J. Nutr..

[bib43] NRC (1989). Diet and Health: Implications for Reducing Chronic Disease Risk. https://www.ncbi.nlm.nih.gov/books/NBK218735/.

[bib44] Olukemi A.R., Abidemi O.O., Abdulsalam S.S. (2016). Functional properties and anti-nutritional factors of some selected Nigerian cereals. Compr. Res. J. Agric. Sci..

[bib45] Özcan M., Bağcı A., Dursun N., Gezgin S., Hamurcu M., Dumlupinar Z., Uslu N. (2017). Macro and micro element contents of several oat (*Avena sativa* L.) genotype and variety grains. Iran. J. Chem. Chem. Eng..

[bib46] Peltonen-Sainio P., Kirkkari A.M., Jauhiainen L. (2003). Characterising strengths, weaknesses, opportunities and threats in producing naked oat as a novel crop for northern growing conditions. Agric. Food Sci..

[bib47] Pinto-Sánchez M.I., Causada-Calo N., Bercik P., Ford A.C., Murray J.A., Armstrong D., Semrad C., Kupfer S.S., Alaedini A., Moayyedi P., Leffler D.A., Verdú E.F., Green P. (2017). Safety of adding oats to a gluten-free diet for patients with celiac disease: systematic review and meta-analysis of clinical and observational studies. Gastroenterol..

[bib48] Rasane P., Jha A., Sabikhi L., Kumar A., Unnikrishnan V.S. (2013). Nutritional advantages of oats and opportunities for its processing as value added foods - a review. J. Food Sci. Technol..

[bib49] Samtiya M., Aluko R.E., Dhewa T. (2020). Plant food anti-nutritional factors and their reduction strategies: an overview. Food Prod. Process. Nutr..

[bib50] Sandberg A.S. (2002). Bioavailability of minerals in legumes. Br. J. Nutr..

[bib51] Sandhu K.S., Godara P., Kaur M., Punia S. (2017). Effect of toasting on physical, functional and antioxidant properties of flour from oat (*Avena sativa* L.) cultivars. J. Saudi Soc. Agric. Sci..

[bib52] Sangwan S., Singh R., Tomar S.K. (2014). Nutritional and functional properties of oats: an update. J. Innov. Biol..

[bib53] Savage G.P., Vanhanen L., Mason S.M., Ross A.B. (2000). Effect of cooking on the soluble and insoluble oxalate content of some New Zealand foods. J. Food Compos. Anal..

[bib54] Siener R., Hönow R., Voss S., Seidler A., Hesse A. (2006). Oxalate content of cereals and cereal products. J. Agric. Food Chem..

[bib55] Sterna V., Zute S., Brunava L. (2016). Oat grain composition and its nutrition benefice. Agric. Agric. Sci. Proced..

[bib56] Van den Broeck H., Londono D., Timmer R., Smulders M., Gilissen L., Van der Meer I. (2015). Profiling of nutritional and health-related compounds in oat varieties. Foods.

[bib57] Whitney E.N., Rolfes S.R. (2011). Understanding Nutrition: Australia and New Zealand.

[bib58] WHO (1996). World Health Organization. Trace Elements in Human Nutrition and Health. https://apps.who.int/iris/handle/10665/37931.

[bib59] WHO (2005). Vitamin and Mineral Requirements in Human Nutrition. https://apps.who.int/iris/handle/10665/42716.

[bib60] WHO (2012). Guideline: Potassium Intake for Adults and Children. https://www.who.int/publications/i/item/9789241504829.

[bib61] Youssef M.K.E., Nassar A.G., EL–Fishawy F.A., Mostafa M.A. (2016). Assessment of proximate chemical composition and nutritional status of wheat biscuits fortified with oat powder. Assiut J. Agric. Sci*.*.

[bib62] Zhou M.X., Glennie Holmes M., Robards K., Helliwell S. (1998). Fatty acid composition of lipids of Australian oats. J. Cereal. Sci..

